# An investigation into the therapeutic effects of statins with metformin on polycystic ovary syndrome: a meta-analysis of randomised controlled trials

**DOI:** 10.1136/bmjopen-2014-007280

**Published:** 2015-03-27

**Authors:** Jie Sun, Yang Yuan, Rongrong Cai, Haixia Sun, Yi Zhou, Pin Wang, Rong Huang, Wenqing Xia, Shaohua Wang

**Affiliations:** Department of Endocrinology, The Affiliated ZhongDa Hospital of Southeast University, Nanjing, People's Republic of China

**Keywords:** GENERAL MEDICINE (see Internal Medicine)

## Abstract

**Objectives:**

To investigate the therapeutic effects of statins with metformin on polycystic ovary syndrome (PCOS).

**Settings:**

Endocrinology department.

**Participants:**

MEDLINE, EMBASE and Cochrane Central Register of Controlled Trials were searched until October 2014. Studies comparing statins and placebo, as well as the combination of statins and metformin and metformin alone, were included in the analysis.

**Interventions:**

Data were independently extracted by two researchers; any convergence was resolved by a third reviewer.

**Primary and secondary outcome measures:**

The following properties were extracted from the qualified trials to identify the effects of statins: clinical variables, metabolic characteristics, hormone outcomes, sign of inflammation, glucose parameters and insulin outcomes.

**Results:**

Data from four trials comparing statin and metformin with metformin alone were analysed. The combination of statins and metformin decreases the levels of C reactive protein (standardised mean difference (SMD) −0.91; 95% CI −1.81 to −0.02; p=0.046), triglyceride (SMD −1.37; 95% CI −2.46 to −0.28; p=0.014), total cholesterol (SMD −1.28; 95% CI −1.59 to −0.97; p=0.000) and low-density lipoprotein (LDL) cholesterol (SMD −0.74; 95% CI −1.03 to −0.44; p=0.000). However, the combined therapy fails to reduce fasting insulin (SMD −0.92; 95% CI −2.07 to 0.24; p=0.120), homeostasis model assessment of insulin resistance (SMD −1.15; 95% CI −3.36 to 1.06; p=0.309) and total testosterone (SMD −1.12; 95% CI −2.29 to 0.05; p=0.061). Analysis of the five trials comparing statin with placebo shows that statin monotherapy reduces LDL-cholesterol, triglyceride and total cholesterol.

**Conclusions:**

Combined statin and metformin therapy can improve lipid and inflammation parameters, but cannot effectively improve insulin sensitivity and reduce hyperandrogenism in women with PCOS. A large-scale randomised controlled study must be conducted to ascertain the long-term effects of the therapy.

**Strengths and limitations of this study**

- No consensus has been reported on routinely co-administering statins with metformin among women with polycystic ovary syndrome (PCOS). Our study investigates the therapeutic effects of statins with metformin on PCOS. Interpretation of the data presented in this meta-analysis presents some limitations.
- First, we did not test the publication bias because a small number of clinical studies were included. Owing to this reason, we included a well-designed RCT by Raja-Khan *et al*^15^ with one woman using oral contraceptive pill (OCP), and two trials^9^ ^19^ with no information about OCP administration. The metabolic outcomes potentially affected by the OCP’s use could not be fully excluded, although the remaining six trials exclude the patients who used OCPs within 3–6 months before enrolment.
- Second, studies exhibit significant heterogeneity. The nine studies included used different diagnosis criteria for PCOS, leading to different types of participants recruited.
- Third, different types of statins were used, including lipophilic and hydrophilic statins, which could have had adverse effects on glucose metabolism.
- Fourth, the baseline characteristics of the participants in the trials differ in terms of age, body mass index, ethnicity, type of statins used, drug dosage, methodologies and follow-up duration, thus affecting the results. Additionally, only studies reported in English language were included in this meta-analysis.

## Introduction

Polycystic ovary syndrome (PCOS) is one of the most common heterogeneous endocrine disorders and is characterised by obesity, menstrual irregularity, infertility and hyperandrogenemia; PCOS affects 5–10% of reproductive-age women.^1^ PCOS is also related to hyperlipidaemia, hyperinsulinaemia, insulin resistance (IR), systemic inflammation and endothelial dysfunction; hence, PCOS increases the risk of gestational diabetes, type 2 diabetes and cardiovascular morbidity.^2^

IR with hyperinsulinaemia is common in lean and obese women with PCOS; this condition is associated with women's reproductive abnormalities, including fetal macrosomia, polyhydramnios, operative delivery, high perinatal mortality and neonatal metabolic complications.^3^ Approximately 40% of women with PCOS exhibit glucose intolerance.^4^ The optimal therapy for PCOS should improve insulin sensitivity through lifestyle and drug intervention.

Metformin has been commonly used to increase insulin sensitivity in women with PCOS. The predicted and confirmed benefits of metformin include decreased hepatic glucose, decreased testosterone level and high peripheral insulin sensitivity.^5^ However, several trials have failed to observe any significant improvement in lipid profile after metformin treatment.^6^ ^7^ The use of statins has recently emerged as a novel therapeutic approach to PCOS.^8^ Treatments using statins, and combined statins and metformin can effectively improve IR, fasting insulin (F-INS), insulin sensitivity index,^9^ hyperandrogenemia,^10^ hirsutism, acne,^11^ testosterone^10^ and decreasing C reactive protein (CRP).^10^ ^11^ Administering atorvastatin pretreatment for 3 months followed by metformin in patients with PCOS improves insulin and homeostasis model assessment of IR (HOMA-IR) indices and reduces CRP level but does not improve the lipid profile compared with placebo treatment; hence, atorvastatin pretreatment enhances the effects of metformin in improving IR, whereas inflammatory markers are not affected by decreased low-density lipoprotein cholesterol (LDL-C) and total cholesterol (TC) after cessation of atorvastatin.^12^ A study showed that statins improve chronic inflammation and lipid profile but deteriorate insulin sensitivity, as indicated by the increased levels of insulin and insulinogenic indices; hence, women with PCOS present an increased risk of type 2 diabetes mellitus and cardiovascular diseases.^13^ Statin therapy is a controversial issue in treatment of PCOS. Sample sizes in published trials are small. The current meta-analysis aims to confirm the therapeutic effects of statins, and statins with metformin, on metabolic and hormone outcomes, particularly insulin sensitivity, among women with PCOS and to eventually elucidate the potential mechanism.

## Materials and methods

### Search strategy and selection criteria

MEDLINE, EMBASE and Cochrane Central Register of clinical trials were systematically searched monthly until October 2014 to obtain pertinent studies. The following combinations of search terms were used: “(PCOS OR polycystic ovary syndrome OR ovary polycystic disease OR ovary syndrome OR hyperandrogenemia) and (statin OR lipidemic-modulating OR lipid lowering drugs OR HMG-CoA reductase inhibitor OR atorvastatin OR fluvastatin OR lovastatin OR pravastatin OR rosuvastatin OR simvastatin).”

Randomised controlled trials in humans and studies reported in English language were included in this meta-analysis. Two independent reviewers assessed studies performed in patients diagnosed with PCOS and excluded those conducted in patients with other diseases. No limit was assigned for PCOS diagnosis. Trials comparing statins with oral contraceptive pills or with other types of statins were excluded. Any divergence was resolved through discussion with a third reviewer.

### Data sources

The following properties were extracted from the qualified trials to identify the effects of statins: clinical variables (age and body mass index (BMI)), metabolic characteristics (LDL-C, high-density lipoprotein cholesterol (HDL-C), TC, triglyceride (TG)), hormone outcomes (total testosterone, androstenedione, dehydroepiandrosterone sulfate (DHEAS), sex hormone-binding globulin (SHBG), free androgen index (FAI), follicle-stimulating hormone (FSH) and luteinising hormone (LH)), sign of inflammation (CRP), glucose parameters (fasting blood glucose (FBG)) and insulin outcomes (F-INS and HOMA-IR). A second reviewer checked the data for accuracy.

### Statistical analysis

Study quality was assessed using Jadad score in table 1. The outcomes presented as mean value and SD were statistically analysed using Stata V.11.0. Weighted mean differences with 95% CIs were selected to describe the mean differences in statin treatment; statistical heterogeneity among the trials was calculated using I² statistics (with 95% CIs) derived from Cochran's Q(100×(Q–df/Q)) (χ^2^ test).^14^ Random-effect models, instead of fixed-effect models, were selected to more effectively assess the average effect. p Values lower than 0.05 and 95% CI without unity were considered statistically significant. We performed sensitive analysis by removing one trial. Funnel plots and Egger's test were undertaken to test for publication bias.

**Table 1:** Characteristics of included studies

Author	Year	Country	Follow-up	Diagnosis of PCOS	Patient selection	Jadad score	n	Intervention
Puurunen	2013	Finland	6 months	ESHRE	Randomised, double blind	6	15	Atorvastatin 20 mg/day qd
							13	Placebo
Raja-Khan	2010	USA	6 weeks	NIH	Randomised, double blind	5	9	Atorvastatin 40 mg/day qd
							11	Placebo
Sathyapalan*	2009	UK	12 weeks	ESHRE	Randomised, double blind	6	19	Atorvastatin 20 mg/day qd
							18	Placebo
Sidika	2013	USA	3 months	ESHRE	Randomised	4	18	Metformin 850 mg/day bid+simvastatin 20 mg/d qd
							20	Metformin 850 mg/day bid
Celik	2012	Turkey	12 weeks	ESHRE/ASRM	Randomised	6	18	Metformin 2000 mg/day qd+rosuvastatin 10 mg/day qd
							20	Metformin 2000mg/day
Kazerooni	2010	Iran	12 weeks	ESHRE/ASRM	Randomised, double blind	5	42	Metformin 500 mg tid+simvastatin 20 mg/day qd
							42	Metformin 500 mg tid
Sathyapalan	2009	UK	6 months	ESHRE	Randomised, double blind	5	19	Atorvastatin 20 mg/day qd 3 months, metformin 500 mg tid 3 months
							18	Placebo 3 months, metformin 500 mg tid 3 months

*Sathyapalan, included three data sets that have been separately analysed.

ASRM, American Society for Reproductive Medicine; bid, two times a day; ESHRE, the Rotterdam European Society for Human Reproductive and Embryology; NIH, the 1990 National Institutes of Health; PCOS, polycystic ovary syndrome; qd, four times a day; tid, three times a day.

## Results

**Figure 1:**
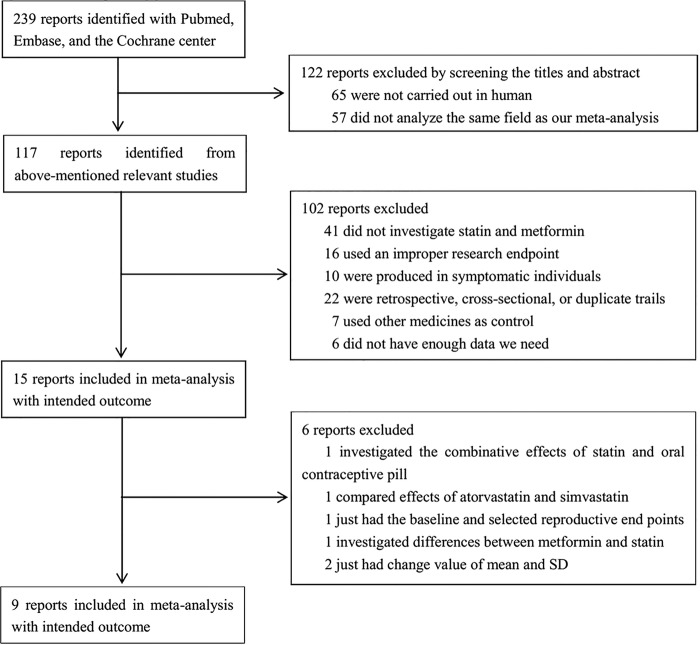
Flow chart.

Initial research yielded 239 studies. We discarded 122 studies after screening the titles and abstracts. After reading the full text of the remaining studies, we further excluded 102 articles. Among the remaining 15 studies, 6 were excluded because they did not present mean and SD values (figure 1). We investigated six trials from unpublished data with a formal question, but we received rejection or no reply. Finally, nine studies that satisfied the predefined criteria were included for meta-analysis. Among these trials, five studies compared statins with placebo,^13^ ^15–18^ and four compared the combination of statins and metformin with metformin alone^9^ ^12^ ^19^ ^20^ (table 1).

### Meta-analysis 1: statins versus placebo

#### Lipid metabolism indicators

**Figure 2:**
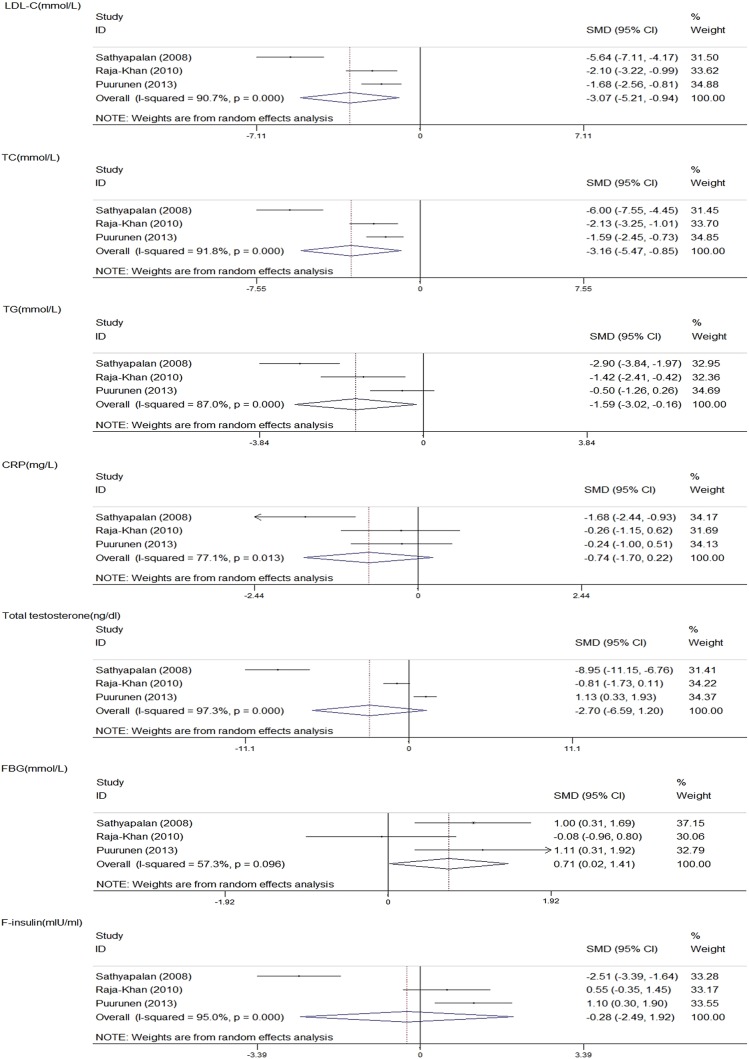
Meta-analysis 1: Statins versus placebo (CRP, C reactive protein; FBG, fasting blood glucose; F-insulin, fasting insulin; LDL-C, low-density lipoprotein cholesterol; SMD, standardised mean difference; TC, total cholesterol; TG, triglyceride).

Among the five studies comparing the effects of statin and placebo, three detected data on the change in LDL-C, HDL-C, TG and TC.^13^ ^15^ ^16^ Statins can significantly lower LDL-C (standardised mean difference (SMD) −3.07; 95% CI −5.21 to −0.94; p=0.005), TC (SMD −3.16; 95% CI −5.47 to −0.85; p=0.007) and TG (SMD −1.59; 95% CI −3.02 to −0.16; p=0.029). Substantial heterogeneities were observed in LDL-C (I²=90.7%, p=0.000), TC (I²=91.8%, p=0.000) and TG (I²=87.0%, p=0.000). HDL-C remained constant (SMD, −0.06; 95% CI −0.49, 0.36; p=0.766) with no heterogeneity detected (I²=0.0%, p=0.827; figure 2).

#### Inflammatory metabolism indicators

Of the five trials in the statin and placebo group, three provided data on the change in CRP.^13^ ^15^ ^16^ The pooled effect demonstrated that statins evidently differ with placebo (SMD −0.74; 95% CI −1.70 to 0.22; p=0.131) with significant heterogeneity (I²=77.1%, p=0.013; figure 2).

#### Hormone metabolism indicators

Of the five trials in the statin and placebo group, three provided data on the change in total testosterone,^13^ ^15^ ^17^ androstenedione and DHEAS, while two provided data on the change in SHBG and FAI. No reduction was observed in the following: total testosterone (SMD −2.70; 95% CI −6.59 to 1.20; p=0.174), androstenedione (SMD −0.50; 95% CI −1.72 to 0.72; p=0.423), DHEAS (SMD −0.60; 95% CI −1.20 to 0.00; p=0.051), SHBG (SMD 0.93; 95% CI −1.65 to 3.51; p=0.481) and FAI (SMD −4.55; 95% CI −15.48 to 6.37; p=0.414). Heterogeneities were detected in total testosterone (I²=97.3%, p=0.000), androstenedione (I²=85.5%, p=0.001), SHBG (I²=95.3%, p=0.000) and FAI (I²=98.6%, p=0.000). However, no heterogeneity was detected in the level of DHEAS (I²=45.2%, p=0.161; figure 2).

#### Glucose metabolism indicators

Three trials were identified among the included five trials.^13^ ^15^ ^18^ The superiority of statins to placebo in reducing F-INS was not confirmed (SMD −0.28; 95% CI −2.49 to 1.92; p=0.800) and heterogeneity existed across the studies (I²=95.0%, p=0.000). The pooled effect of statins showed an increased FBG level (SMD 0.71; 95% CI 0.02 to 1.41; p=0.044), but without significant heterogeneity (I²=57.3%, p=0.096; figure 2).

### Meta-analysis 2: statin+metformin versus metformin

#### Lipid metabolism indicators

**Figure 3:**
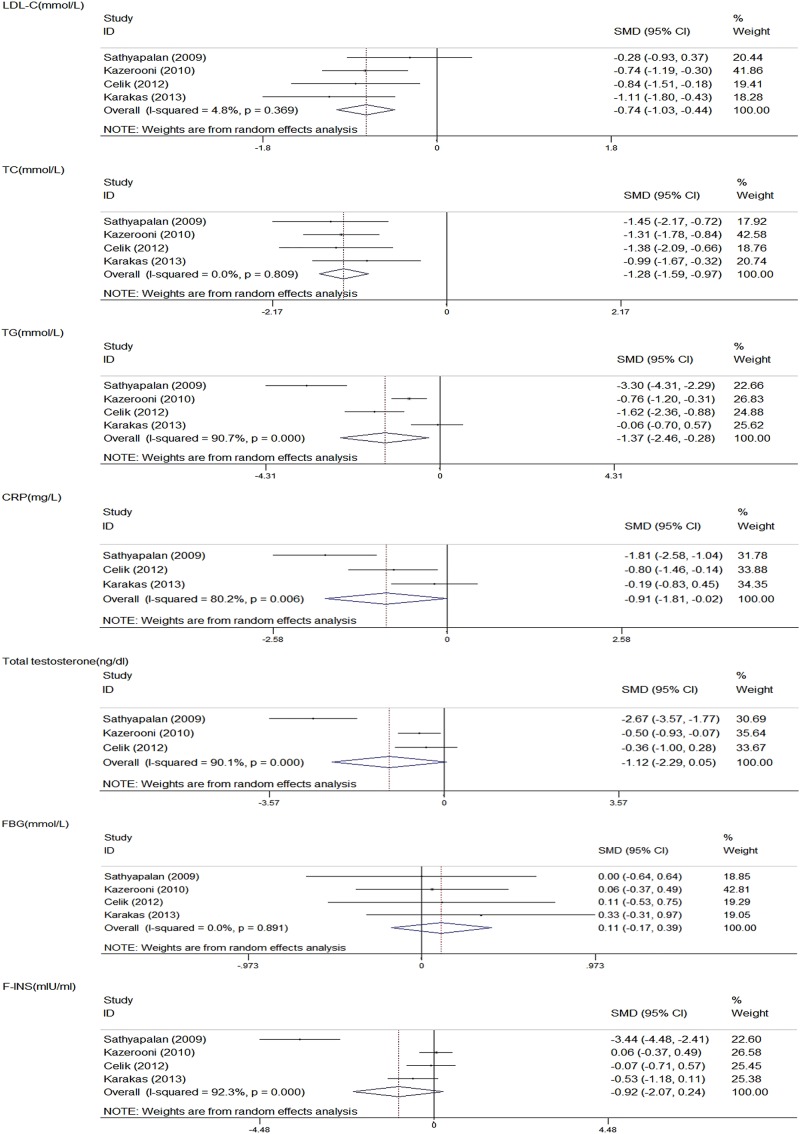
Meta-analysis 2: Statin+metformin versus metformin (CRP, C reactive protein; FBG, fasting blood glucose; F-INS, fasting insulin; LDL-C, low-density lipoprotein cholesterol; SMD, standardised mean difference; TC, total cholesterol; TG, triglyceride).

All the four studies detected data on the change in LDL-C, HDL-C, TG and TC.^9^ ^12^ ^19^ ^20^ The combined statin plus metformin reduced LDL-C (SMD −0.74; 95% CI −1.03 to −0.44; p=0.000), TC (SMD −1.28; 95% CI −1.59 to −0.97; p=0.000) and TG (SMD −1.37; 95% CI −2.46 to −0.28; p=0.014). Substantial heterogeneity was observed in TG (I²=90.7%, p=0.000), whereas no heterogeneity was detected in LDL-C (I²=4.8%, p=0.369) and TG (I²=0.0%, p=0.809). HDL-C was not significantly decreased by the treatment (SMD −0.04; 95% CI −0.64 to 0.56; p=0.884), but exhibited significant heterogeneity (I²=76.1%, p=0.006; figure 3).

#### Inflammatory metabolism indicators

Of the four trials comparing statin and metformin versus metformin group, three provided data on the change in CRP.^12^ ^19^ ^20^ After the meta-analysis, the combined treatment remarkably decreased the CRP level (SMD −0.91; 95% CI −1.81 to −0.02; p=0.046) and exhibited heterogeneity across the studies (I²=80.2%, p=0.006; figure 3).

#### Hormone metabolism indicators

Of the four trials comparing statin and metformin versus metformin group, three provided data on the change in total testosterone,^9^ ^12^ ^19^ while two provided data on the change in DHEAS, FSH and LH.^9^ ^19^ The combined therapy failed to reduce total testosterone (SMD −1.12; 95% CI −2.29 to 0.05; p=0.061), DHEAS (SMD −0.40; 95% CI −1.13 to 0.33; p=0.282), FSH (SMD −0.16; 95% CI −0.52 to 0.19; p=0.375) and LH (SMD −1.39; 95% CI −4.18 to 1.41; p=0.331). Heterogeneities were also observed in total testosterone (I²=90.1%, p=0.000) and LH (I²=97.5%, p=0.000), whereas no substantial heterogeneity was observed in DHEAS (I²=71.1%, p=0.063) and FSH (I²=0.0%, p=0.697; figure 3).

#### Glucose metabolism indicators

All the four studies assessed FBG and F-INS.^9^ ^12^ ^19^ ^20^ The pooled effect of the combined treatment showed no significant difference in F-INS (SMD −0.92; 95% CI −2.07 to 0.24; p=0.120) and HOMA-IR (SMD −1.15; 95% CI −3.36 to 1.06; p=0.309), but showed high heterogeneities (I²=92.3%; p=0.000 and I²=94.3%; p=0.000) compared with statin therapy. Moreover, the combined therapy did not evidently affect the level of FBG (SMD 0.11; 95% CI −0.17, 0.39; p=0.443). The heterogeneity across the trials was low (I²=0.0%, p=0.891), indicating that most variations were incidental (figure 3).

### Sensitivity analysis

One trial was removed for sensitivity analysis. The remaining trials still present similar results in heterogeneity and pooled effect. Publication bias was assessed using the funnel plot (online supplementary file 1 ‘funnel plot’). Overall, different subject profiles may explain the heterogeneity observed.

## Discussion

Our meta-analysis shows that the combined therapy of statins and metformin fails to improve insulin sensitivity and hyperinsulinaemia but decreases the serum levels of LDL-C, TC, TG and CRP; these findings are consistent with the therapeutic effect of statin therapy on women with PCOS. The combined therapy does not increase the FBG level; however, statin alone can increase the FBG level.

The lipid-lowering effect of statins administered with or without metformin in women with PCOS remains ambiguous. This finding is in accordance with the meta-analysis performed by Gao *et al*.^21^ Unlike previous meta-analyses, our study demonstrates that statins, and combined statins and metformin, do not beneficially affect serum testosterone and insulin sensitivity. Gao *et al* proved that the use of statins alone reduces serum testosterone, and the combined statin and metformin therapy improves serum testosterone and IR. A possible explanation for this discrepancy could be attributed to the different inclusion criteria used in these studies. Our study selected trials with data expressed as mean and SD, whereas that of Gao *et al* included trials with data expressed as changed value of mean and SD. This standard was also used to include more trials, resulting in a more reliable pooled effect. Moreover, the study of Kazerooni *et al*^9^ assessed the effect of the combination of simvastatin and metformin on biochemical parameters compared with combined metformin and placebo. This study was included in the second step of the present meta-analysis to compare statins and with the combined therapy. However, Gao selected this trial to compare the therapeutic effects between statins and placebo.

Although statin treatment improves insulin sensitivity^22^ ^23^ in patients with PCOS,^12^ increasing evidence shows that this therapy negatively affects glucose metabolism in hypercholesterolaemic patients with PCOS.^24^ Animal experiments showed that atorvastatin can reduce insulin sensitivity and impair glucose tolerance in rats.^25^ Furthermore, a human trial demonstrated increased insulin secretion after 6 weeks of statin therapy in women with PCOS.^15^ Our meta-analysis found that statins fail to improve F-INS and HOMA-IR in statins alone or in combination with metformin. This finding may be due to the following reasons. First, statins may damage endothelial function through loss of the protective anti-angiogenic and anti-proliferative effects of adiponectin, resulting in impaired insulin sensitivity.^26^ Second, statins decrease the levels of cholesterol mediated by the farnesoid X receptor (FXR), the deficiency of which is related to IR.^27^ The activation of FXR can lower the levels of glucose-6-phosphatase, reduce phosphoenolpyruvate carboxykinase in gluconeogenesis, and increase glycogen synthesis.^28^ Hence, induced IR caused by statin therapy may be related to the low expression of FXR.^29^ Third, lipophilic statins are possibly absorbed by extra-hepatic cells; these statins can deregulate cholesterol metabolism, thus attenuating β-cell function and deteriorating IR.^30^ Similarly, we also determined that statins with and without metformin cannot improve total testosterone level. In parallel with our meta-analysis, several studies suggest that statins do not affect the level of total testosterone in postmenopausal women.^31^ Primary activities possibly occur in the ovary, and statins fail to decrease the level of testosterone in postmenopausal women because of the extraovarian androgens.^31^ Moreover, not all statins can suppress gonadal hydroxymethylglutaryl coenzyme A reductase at specified doses and cholesterol sufficiently maintains testosterone synthesis.^32^ Finally, the baselines of the hormones differ from the trials, providing inaccurate comparison of terminal values.

### Limitations

Interpretation of the data presented in this meta-analysis presents some limitations. First, we did not test the publication bias because a small number of clinical studies were included. Owing to this reason, we included a well-designed RCT by Raja-Khan *et al*,^15^ with one woman using oral contraceptive pill (OCP) and two trials^9^ ^19^ with no information about OCPs administration. The metabolic outcomes potentially affected by the OCPs use could not be fully excluded, although the remaining six trials exclude the patients who used OCPs within 3–6 months before enrolment. Second, studies exhibit significant heterogeneity. The nine studies included used different diagnosis criteria for PCOS, leading to different types of participants recruited. Third, different types of statins were used, including lipophilic and hydrophilic statins, which could have had adverse effects on glucose metabolism.^33–35^ Fourth, the baseline characteristics of the participants in the trials differ in terms of age, BMI, ethnicity, type of statins used, drug dosage, methodologies and follow-up duration, thus affecting the results. Additionally, only studies reported in English language were included in this meta-analysis.

### Conclusions

In spite of these limitations, our meta-analysis shows that statin therapy, and combined statin and metformin therapy, can improve lipid and inflammation parameters, as well as effectively prevent the risk of cardiovascular diseases among women with PCOS. Nevertheless, the evidence on routine statin therapy in these patients is still limited. Statins alone or combined with metformin cannot effectively improve insulin sensitivity and reduce hyperandrogenism. Additionally, statins may slightly increase FBG in women with PCOS. A large-scale randomised controlled study should be performed to ascertain the long-term effects of statins.
